# Coffee By-Products as Sustainable Novel Foods: Report of the 2nd International Electronic Conference on Foods—“Future Foods and Food Technologies for a Sustainable World”

**DOI:** 10.3390/foods11010003

**Published:** 2021-12-21

**Authors:** Dirk W. Lachenmeier, Steffen Schwarz, Jörg Rieke-Zapp, Ennio Cantergiani, Harshadrai Rawel, María Angeles Martín-Cabrejas, Maria Martuscelli, Vera Gottstein, Simone Angeloni

**Affiliations:** 1 Chemisches und Veterinäruntersuchungsamt (CVUA) Karlsruhe, Weissenburger Strasse 3, 76187 Karlsruhe, Germany; vera.gottstein@cvuaka.bwl.de; 2 Coffee Consulate, Hans-Thoma-Strasse 20, 68163 Mannheim, Germany; schwarz@coffee-consulate.com; 3 Rubiacea Research and Development GmbH, Hans-Thoma-Strasse 20, 68163 Mannheim, Germany; joerg.rieke_zapp@yahoo.de; 4 Académie du Café, Rue du Village 17, 1803 Chardonne, Switzerland; ennio.cantergiani@gmail.com; 5 Institute of Nutritional Science, University of Potsdam, Arthur-Scheunert-Allee 114-116, 14558 Nuthetal, Germany; rawel@uni-potsdam.de; 6 Department of Agricultural Chemistry and Food Science, Universidad Autónoma de Madrid, 28049 Madrid, Spain; maria.martin@uam.es; 7 Institute of Food Science Research, CIAL (UAM-CSIC), 28049 Madrid, Spain; 8 Faculty of Bioscience and Technology for Food, Agriculture and Environment, University of Teramo, Via R. Balzarini 1, 64100 Teramo, Italy; mmartuscelli@unite.it; 9 Department of Food Chemistry and Phytochemistry, Karlsruhe Institute of Technology (KIT), Adenauerring 20A, 76131 Karlsruhe, Germany; 10 School of Pharmacy, University of Camerino, Via Sant’Agostino 1, 62032 Camerino, Italy; simone.angeloni@unicam.it

**Keywords:** coffee by-products, sustainable world, coffee leaves, coffee flower, coffee cherry, coffee pulp, cascara, parchment, coffee silverskin, beverages, coffee grounds, food safety, novel food

## Abstract

The coffee plant *Coffea* spp. offers much more than the well-known drink made from the roasted coffee bean. During its cultivation and production, a wide variety of by-products are accrued, most of which are currently unused, thermally recycled, or used as fertilizer or animal feed. Modern, ecologically oriented society attaches great importance to sustainability and waste reduction, so it makes sense to not dispose of the by-products of coffee production but to bring them into the value chain, most prominently as foods for human nutrition. There is certainly huge potential for all of these products, especially on markets not currently accessible due to restrictions, such as the novel food regulation in the European Union. The by-products could help mitigate the socioeconomic burden of coffee farmers caused by globally low coffee prices and increasing challenges due to climate change. The purpose of the conference session summarized in this article was to bring together international experts on coffee by-products and share the current scientific knowledge on all plant parts, including leaf, cherry, parchment and silverskin, covering aspects from food chemistry and technology, nutrition, but also food safety and toxicology. The topic raised a huge interest from the audience and this article also contains a Q&A section with more than 20 answered questions.

## 1. Introduction

When talking about coffee, people usually think about the drink from the roasted bean, but the coffee plant offers much more than the roasted bean. Various by-products are accrued during the cultivation of the plant. Currently, these are often called “coffee waste products”, which should be changed in today’s sustainable societies because they can be used as food and for other applications.

**Figure 1:**
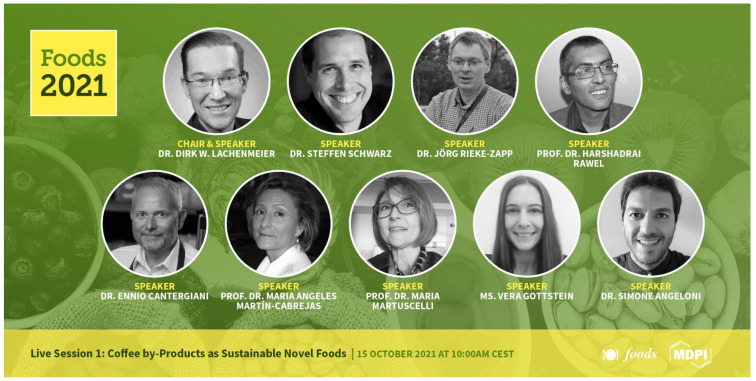
Speakers of the Foods 2021 Conference live session 1 on coffee by-products.

This article summarizes the major outcomes of the first live session of The 2nd International Electronic Conference on Foods—“Future Foods and Food Technologies for a Sustainable World” entitled “Coffee By-products as Sustainable Novel Foods”. Dirk W. Lachenmeier chaired the conference and was also a speaker. The participating experts were Steffen Schwarz, Jörg Rieke-Zapp, Ennio Cantergiani, Harshadrai Rawel, María Angeles Martín-Cabrejas, Maria Martuscelli, Vera Gottstein, and Simone Angeloni (Figure 1). The audience of more than 100 participants contributed with questions and comments. The conference session was live streamed on MDPI’s YouTube channel and a video recording is available on the conference webpage (https://foods2021.sciforum.net/#recordings (accessed on 17 December 2021)). The conference evaluation committee chose Simone Angeloni for the Best Speaker Award.

**Figure 2:**
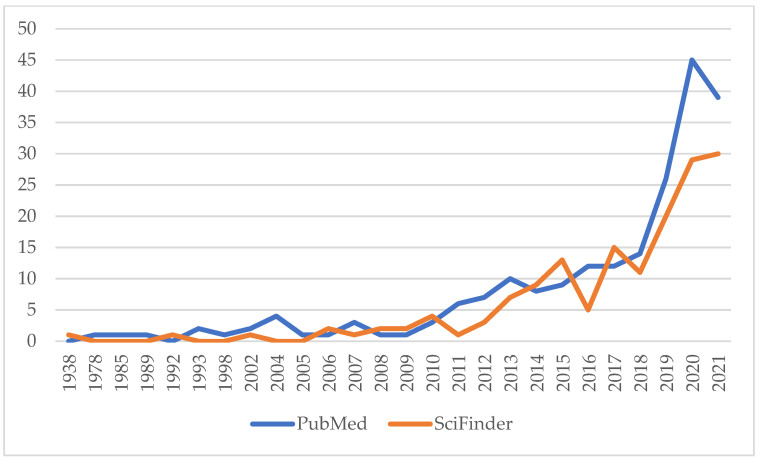
Scientific publications 1938–2021 on coffee by-products. Sources: PubMed, National Library of Medicine, Bethesda, MD, USA; SciFinder^n^, CAS, American Chemical Society (ACS), Columbus, OH, USA. Search term: “coffee by-product” OR “coffee byproduct”, searches conducted 17 December 2021.

It is notable that this session is the first conference session exclusively dedicated to coffee by-products, which received considerably increased research interest over the last few years (Figure 2). Before 2010, only a few papers were published about the topic starting with a rather visionary early report on the applications of coffee by-products published in 1938 by do Amaral, where several of the fields mentioned in this conference were already suggested [1]. Subsequently, only a few articles on the use of coffee pulp as animal feed were published in the 1970s and 1980s [2,3].

This conference session was intended to summarize the science of coffee by-products, but also include practical aspects about how these products might be used and commercialized. Legal issues, such as novel food regulations, are covered, as well as analytical aspects. This article provides a synopsis of the live session, held on 15 October 2021, providing summaries from all speakers, as well as an edited transcript of the Q&A session.

## 2. Summary of Conference Presentations

### 2.1. Steffen Schwarz: An Introduction to Coffee By-Products

**Figure 3:**
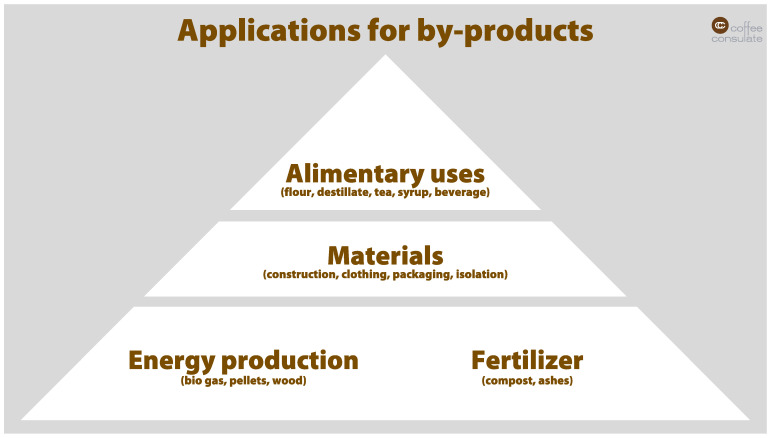
Pyramid model for applications of coffee by-products.

Coffee by-products are far more than by-products, they are products. The seed should even be seen as the by-product of what the coffee plant is producing. The entire coffee production needs to be rethought. Bringing this into the minds of the entire coffee industry can change the life of all coffee farmers. Figure 3 visualizes the potential use of the products. Primarily, the focus should be dietary use. Secondly, they can be used as a material, such as construction material, for clothing, for packaging, or for isolation. Then finally, the products could be used for energy production as a source for biogas, for pellets, or for wood production.

Coffee leaves (see Section 2.3) have been traditionally used as a tea-like beverage in Ethiopia, also in Yemen, and in some other parts of Southeast Asia [4,5]. Coffee flowers are currently not widely recognized as a by-product. Due to their flavor between rosebud and jasmine, they could be applied for bakery and sweet products

The coffee husk, better known as cascara (see Section 2.4), is a product coming out of dry processing from the coffee cherry, which can be used as a tea. For bakery products, it can be turned into flour or syrups. The coffee pulp is the product from the wet processing (see Section 2.5). So both are very close. Applications include distilling and different forms of beverages, syrups and purees [6].

The parchment may be used as a packaging material. It can also be used for isolation. However, there are also food uses of the parchment (see Section 2.6). Moreover, it can be turned into ashes to be used as a fertilizer.

The silver skin emerges in masses during roasting [6]. Historically it had been used as animal feed in Germany, but it can be used for bakery products or as protein bars. It has a valuable combination of fibers and proteins (see Section 2.7, Section 2.8, Section 2.9). Finally, coffee wood can be produced when the trees have reached the end of their life.

More details about the coffee by-products are provided in the proceedings article [7] and a previous review article [5].

### 2.2. Dirk Lachenmeier: An Update on Sustainable Valorization of Coffee By-Products as Novel Foods within the European Union

In the European Union (EU), novel foods (i.e., foods not marketed before 1997) need approval before they can be put on the market. During the approval procedure, the products must be evaluated for safety and toxicological aspects. According to the EU novel food catalog, roasted coffee beans are not novel. However, dried *Coffea* berries (husk or cascara) are considered novel. There are two pathways for the approval of novel foods: a full application or a notification for a traditional food from a third country. Using the latter possibility, coffee leaf infusions have already been approved since 2020. The downside from the notification as a traditional food is that only the traditional uses are approved, in this case the infusion. All other uses would still need a full application.

The novel food approval process for coffee cherry materials such as husks, cascara, dried or fresh cherries, mucilage, and pulp is currently ongoing. There are several applications as traditional food and one full application for coffee cherry. The European Food Safety Authority (EFSA) already published opinions on two of the notifications and did not raise any safety objections against placing the coffee cherry on the market in the EU. The last step of the process, the implementing regulation, is currently still pending and is expected at the end of 2021.

For the silverskin, a consultation request is currently determining its novel food status. The history of silverskin consumption is currently difficult to assess because it has always been consumed as a residue in roasted coffee, but not in pure form as such. For the coffee grounds such a consultation was already finished and they were determined as not being novel.

For other by-products, such as the parchment or the flower, no applications are currently going on. A summary of the novel food status of coffee by-products is provided in Table 1.

The next steps, could be to better align industry efforts, e.g., in the form of a consortium that pools the available data and coordinates the applications. Then the traditional uses in third countries should be better explored, e.g., for uses of the flower or other applications of the cherry or the leaf.

Independent of novel food approval, food business operators must always ensure the safety of the products, especially with coffee pulp from wet processes, there is a risk of contamination with mycotoxins, or microbiological contamination.

A more detailed review of the novel food status of the different coffee by-products is available in the proceedings article for this presentation [7]. Regarding analytical aspects of coffee leaf tea, another proceedings article of the Foods2021 conference is available [4].

### 2.3. Jörg Rieke-Zapp: Coffee Leaf Tea. Traditional Beverage or Novel Food?

When considering a coffee plantation, there is wood, there are leaves, and there are twigs. In addition, the removal of these materials constitutes costs. Leaves and twigs constitute approximately 10% to 30% of gross per hectare per year, which is cut off by pruning.

There are traditional preparations of tea available and known in the countries of origin, such as Amertassa (dried and crushed coffee leaves) or Kati (similar as Amertassa but roasted on top).

**Figure 4:**
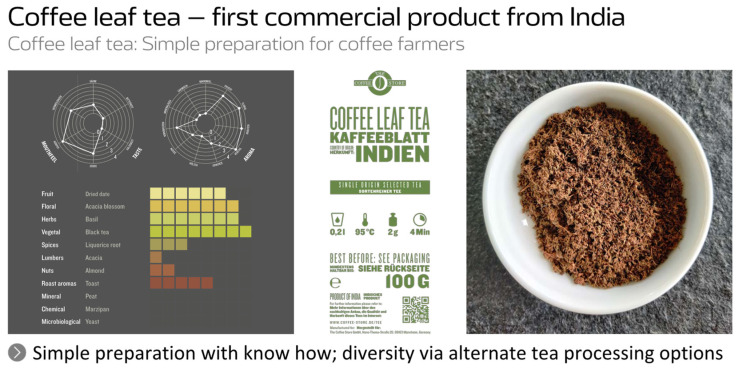
Representation of a commercial coffee leaf tea from India.

According to own experience (Figure 4), it needs some time for farmers to establish a good and simple preparation of coffee leaf tea. They do not want to have a tedious picky product, like traditional *Camellia sinensis* tea. This does not make sense for coffee at all because trees cannot be cut as is done with tea bushes. Therefore, at the end, farmers might want to use everything they cut out. Therefore, they are using a lot larger leaves if compared to tea. Moreover, this works brilliantly fine. Because the interesting thing about coffee leaf tea is that it is not going to be bitter. Therefore, it is not turning bitter even on longer steeping time. It is a preparation that is more or less done with low fermentation like a green tea, providing consumers with a kind of dried date fruit aroma. It has a floral taste. It has an herbal, vegetal black tea note and it is a bit spicy. It is almost like the green tea coming from China or Japan. But just for bitterness, it has almost the character of teas that come from very old tea bushes, which are very sought after.

For processing, all the different possibilities of tea can also be used also for the leaves of coffee. Processing can include more fermentation, more oxidation, etc.

In addition, this is really a benefit for farmers, because they can produce coffee leaf tea or coffee leaf products all year. Therefore, they have income and plant care is refinancing. Farmers may use the drying capacity more efficiently, because the drying capacity is usually not used throughout the year.

Additionally, there is obviously a high market demand because industry wants to have novel beverages with caffeine, and obviously the coffee leaf contains a lot of caffeine. Moreover, it is released like in a standard tea, i.e., a slow release formulation of caffeine. This is attractive because industry is always looking for beverages, which are not bitter, which have a pleasant flavor profile, and, which give some caffeine.

### 2.4. Ennio Cantergiani: Cascara—A Quality Perspective

Switzerland authorized the use of cascara as a traditional food in June 2020, so that this product has already been launched in that country as initial European market. Regarding the quality of cascara, two dimensions may be considered. The first one is food safety, which is ensured by the novel food approval process in Switzerland and the EU (see Section 2.2). The second, and most important part, is the sensory or organoleptic quality of the product. Production processes influence not only sensory quality but also the chemical composition [18]. The two main processes applied in coffee industry greatly affect the product quality. For example, the parchment remains in the cascara from a natural (dry) process. Good agricultural practices for cascara must be established, which are not so different from the good agricultural practices for coffee, but it must be considered that clean cascara is needed. A long drying step is preferred because it develops the color and the fruity flavors, so mechanical dryers are not giving good results. Some producers and farmers have some freezing rooms and store the cascara in the freezing room. This investment can give real benefit. Then packaging and storage are relevant. Vacuum packaging is always preferred, but it costs a lot.

The challenges for farmers include the need for more drying beds, because they are drying green beans and cascara in parallel. They also need additional workers because they must treat green coffee and coffee cherries.

Regarding cupping of cascara, black bowls are preferred to avoid influences by the color. A sensory vocabulary for cascara is currently emerging based on what exists for coffee. With cascara, defects can also often be detected, e.g., moldy, seafood, woody, oysters, fishy, iodine. Green and vegetal were also some characteristics of many cascaras, but the best one has a clean fruity character.

Until now, there have been only a few scientific publications on volatile compounds in cascara [19], so there are still challenges remaining characterizing good and defective cascaras.

The good agricultural practices must be better defined, more volatile analysis to better characterize good cascara must be performed, the cupping protocol needs to be improved and the flavor wheel is to be refined. All these could culminate in an international standard for cascara.

### 2.5. Harshadrai Rawel: Wet-Coffee Processing Production Wastes: Quality, Potentials, and Valorization Opportunities

Wet processing of coffee produces a large amount of wastewater, which is a cause of environmental pollution. Nevertheless, the wastewater also contains some interesting components, it also contains a biological load, and it contains numerous components, which are toxic to the environment. There is the possibility of always using fresh water or recycling the water. Analytical results show that many compounds from coffee cherry can be found in wastewater, e.g., total phenolics and high antioxidant capacity. There are also differences in the composition of the organic acids, for example malic acid. The batch process produces high acidity and high biological load. This will create long-term problems if the water is not properly treated. Finally, many proteins and free amino acids are recovered, which come from microbial organisms, their metabolic products, but also from the coffee cherry itself. Hence, nutritionally, the water could be interesting. Certain secondary plant metabolites, especially phenolic compounds, interact with proteins and these proteins are modified during processing [20,21].

Our challenge is to try improving the quality of products and to increase the potential of wastewater, by recovering components, which are interesting. Active carbon prepared from coffee husk, parchment or other by-products could be applied for cleaning the wastewater. The parchment can also bind and remove several compounds. Hence, it could be possible to use this material to decrease the amount of phenolic compounds in the water [22].

### 2.6. María Angeles Martín-Cabrejas: Revalorization of Coffee Parchment as a Source of Phenolic Compounds and Antioxidant Dietary Fiber

Coffee parchment is one of the most underutilized coffee by-products. It is produced during coffee processing. After harvesting the husk is removed and then the coffee beans are fermented, washed and dried. In this last step, the parchment is removed. The main use of this by-product is for bioenergy production. The parchment is rich in caffeine and phenolic compounds. Heat-assisted extraction can recover the phenolic compounds. Using HPLC characterization of the extract, 13 phenolic compounds were obtained. Another strategy is to use the parchment in flake form or in ground form as a flour. Functional properties were studied, including dietary fiber characterization, antioxidant capacity, polysaccharide composition and some functional properties. The coffee parchment flakes present a high oil-holding capacity also a chelation capacity and relevant hydration properties, as well as capacity for absorption of glucose and inhibition of amylase. The extrusion process, including temperature and moisture settings, of coffee parchment has a significant effect on the chemical and functional properties [23,24,25].

In conclusion, the eco-friendly method usually a heat-assisted aqueous extraction allows to attain compounds from coffee parchment, which includes insoluble dietary fiber. The extrusion process can be used to obtain an ingredient to be incorporated into other foods. Coffee parchment can be used as a promising low-calorie functional ingredient for dietary fiber enrichment in foods. The possibility to regulate blood glucose needs further studies. The aqueous extract of coffee parchment can be a source of phenolic compounds with antioxidant capacity.

### 2.7. Maria Martuscelli: By-Products from the Coffee Industry—Coffee Silverskin

A coffee by-product with a particular interest is the coffee silverskin. It is recovered during roasting by using suction cyclones, and then compacted. Typically, several varieties are combined. Silverskin has a very low water activity, its stability depends on the environmental humidity, and shelf life is prolonged by avoiding water adsorption. It is reasonable to suppose that the high temperature treatment limits the microbial load [26].

The phenolic compounds depend on postharvest processing and roasting conditions, dark, medium or light roasting processes. However, reducing the content of these molecules can positively affect the bitterness, astringency, and cut grass smell sensations that could arise. Due to compounds such as melanoidins, some silverskin can find application as a food dye.

Regarding contaminants, there are reports about pesticides and ochratoxin A. However, analytical artifacts of compounds formed during roasting must be considered, which could be misinterpreted as fragments of pesticides. A typical contaminant found in silverskin is acrylamide [26]. However, reached levels of acrylamide detected in roasted coffee (from which silverskin was collected), were always well below the limits allowed for roasted coffee by European current legislation [27]. The caffeine concentration can be considered safe and was lower than that reported for coffee beverages. The silverskin can improve mineral intake and it is safe for aluminum and nickel levels.

The application of coffee silverskin was tested as a functional ingredient in sponge cake enriched with fibers. Alkaline treatment was performed, so water holding capacity and oil-holding capacity were improved. In particular, the properties of the experimental cake were similar to those of the control. Texture analysis showed slight differences in hardness and deformability, while color was affected by coffee silverskin, leading to a yellow brown color. The silverskin was further tested in newly developed biscuits. In this case, no chemical treatments were provided on coffee silverskin (quality analyses are still ongoing).

Infusion of coffee silverskin is rich in polyphenols similar to green tea and consumer tests show interest in the new infusion. Finally, the role of coffee silverskin against oxidative phenomena was tested in a newly developed chicken meat burger after cooking. The color change in the uncooked burgers can negatively affect the willingness to buy them, but when cooked they look similar to grilled veal meat. Coffee silverskin can solve two problems. The first is limiting the decay of meat foods, especially of poultry origin, the second is the lowering food waste by coffee production. Therefore, it has the potential for being used as a natural additive in meat [28] and potentially become a cheap but valuable ingredient for fibers, minerals, and bioactive molecules uptake.

Finally, considering its chemical composition, silver skin powder could be used for the development of innovative sensors that test the antioxidant activity of foods [29].

### 2.8. Vera Gottstein: Coffee Silverskin: Chemical Characterization with Special Consideration of Dietary Fiber and Heat-Induced Contaminants

The coffee silverskin of *Coffea arabica* and *Coffea canephora* and so-called coffee silverskin pellets were analyzed. Coffee silverskin can have a high amount of dietary fiber of 60% to 80%, low fat content, and a relatively high protein content (Table 2). Due to the high content of dietary fiber, the composition of the fiber was investigated in more detail. Determination of the dietary fiber content showed a comparably high portion of soluble fiber, whereas low molecular weight soluble fiber was not detected. Monosaccharide and methylation analysis confirmed cellulose and indicated xylans in the insoluble dietary fiber fraction, while pectic polysaccharides dominated the soluble dietary fiber fraction. The protein content was about 18 to 22 g/100 g, and all essential amino acids were present in coffee silverskin, whereas fat contents were low. Elemental analysis by inductively coupled plasma mass spectrometry (ICP-MS) showed macro-elements in large amounts, whereas toxic mineral elements were detected only in trace amounts or were absent. Acrylamide was quantified with levels of 24–161 µg/kg. Although 5-hydroxymethylfurfural was detected, its concentration was below the limit of determination. Furfuryl alcohol was not detected [6].

### 2.9. Simone Angeloni: Coffee Silverskin and Spent Coffee Ground: Chemical Characterization and Extract Evaluation

Coffee silverskin is released during the roasting of coffee, while spent coffee ground is the remaining material after coffee brewing. Different extraction methods have been tested to extract and recover bioactive compounds from these two by-products. The obtained extracts were characterized using an HPLC-MS/MS method to detect and quantify 30 bioactive compounds including alkaloids, chlorogenic acids, phenolic acids, flavonoids, iridoids and xanthones [30,31].

Coffee silverskin and spent coffee ground extracts obtained using ethanol/water (70:30) as extraction solvent showed the highest levels of bioactive compounds (4.0% and 7.2% *w*/*w*, respectively). High concentrations of caffeine were observed with levels varying from 1.0% to 5.2% of dry weight of extract. 18 phenolic compounds were detected in coffee silverskin extracts while 20 in those of spent coffee grounds. The data showed that caffeine and chlorogenic acids were the most abundant compounds in all samples followed by phenolic acids. Neuroprotective activity of silverskin and spent coffee extracts against H_2_O_2_-induced damage was evaluated for the first time suggesting for methanol and ethanol/water (70:30) extracts a potential role as protective agents against neurodegeneration due to their ability to counteract oxidative stress and maintain cell viability [31,32].

For the first time, the aroma profile of coffee silverskin has been investigated by GC-O/FID and GCxGC-MS systems, and the intensity of each odor has been ranked by AEDA. Typical coffee odorants, e.g., furaneol, 2-methoxy-4-vinylphenol, 2-methoxyphenol, 2-furfurylthiol, 2,3-butanedione, vanillin and 2-isobutyl-3-methoxypyrazine, occurred in coffee silverskin and some of them possessed similar flavor dilution factors of those found in coffee beans [33].

Therefore, the low cost of these two by-products and their biological activities along with coffee silverskin aroma profiles suggest their possible use as ingredients for the food and pharmaceutical industry [33,34,35].

## 3. Q&A Session

The following section summarizes the highlights from the question and answer session of the conference, including questions that were answered by the expert only in written form in the chat box of the conference system, so that many of them are unavailable in the video recording of the conference.

### 3.1. Are You Aware of Any Current Uses for Coffee Flowers?

There are some coffee flowers being used and they are turned into sweets, they are candied, but one can also use them as dried coffee flowers. They are also used to make tea, and this may have been traditionally done in coffee producing countries. Then there are uses for the cosmetic industry. Coffee flowers are currently used in very small amounts, mainly because they are picked at the wrong time. Many farmers are afraid to pick their flowers, because they incorrectly fear that this leads to no harvest for cherries. For further details on coffee flowers and correct picking, see [7].

### 3.2. How Old Is Coffee Tree Wood When It Is Used as Furniture?

Typically, *C. canephora* wood is applied because it has a more massive stem than *Coffea arabica* wood. The previously applied wood has been about 70 to 100 or 120 years. It is a unique wood with a beautiful color and structure, also see [7].

### 3.3. Do You Have Any Comments on Pectin from Coffee Mucilage or Coffee Husk?

There are high amounts of pectin within the coffee cherry and its pulp compared to other fruits such as sweet cherry or plum. This may cause problems during fermentation for distilling spirits due to methanol formation. For detailed data, see Blumenthal et al. [36].

### 3.4. What Requirements Have You Put in Place with Farmers to Ensure Compliance with Pesticide Legislation for Coffee Leaves? Do You Test Every Batch? What Commodity Do You Refer to in Terms of Maximum Residue Limits (MRL)?

Optimally, organic farming practices should be applied if the leaves or cherries are intended to be used as foods. Otherwise, the practices can be taken from other tea products, such as *Camellia sinensis* tea. If suppliers are uncertain about practices on the farm, testing should also encompass copper for the organic production. Copper may be accumulated in the coffee leaves due to long-term use of copper fungicides, and coffee leaves then showed an extremely high Cu concentration [37]. Pesticides were not detected in organic coffee leaf extracts, but thiametoxam was clearly detected in 50% of coffee leaf extracts harvested from coffee trees grown under conventional conditions [38].

There are certain regulations and maximum limits for pesticides established in most jurisdictions around the world. For pesticide MRL in the EU, the coffee leaf would fall into the group “other leaves/herbs” within the category of “teas, coffee, herbal infusions, cocoa and carobs” [39].

### 3.5. Are There Medical Concerns about Using Coffee Leaves as Herbal Infusion?

No, the EFSA did not have safety concerns regarding the use of the herbal infusion [10]. For some compounds such as caffeine and epigallocatechin gallate, the novel food approval provided maximum limits [4].

### 3.6. Are Coffee Dried Leaves or Cascara Approved for Sale in Markets?

The products are traditionally available in many countries worldwide. In markets where novel food regulations apply, approval might be necessary. For example in the EU, coffee leaf tea infusion is now approved [9], cascara is still pending (see Table 1). Cascara has already been authorized in Switzerland.

### 3.7. What Is the Caffeine Content in Coffee Leaf Infusion?

This depends on the coffee species (*C. arabica*, *C. canephora*, *C. liberica*, etc.) and of course the concentration (how many grams per cup). It can be compared to the amount of coffee. Therefore, it is higher than in tea. The EU novel food approval allows a maximum caffeine concentration of 80 mg/L in coffee leaf infusion [4,9].

### 3.8. Do You Have Any Suggestions on Harvesting Coffee Leaves for Making the Infusion? Some Say That When Coffee Is about to Harvest, Some Number of Leaves Are Still Needed to Keep the Quality of Coffee Cherry Quality

Of course, removing most or even all the leaves would cause loss of harvest. However, farmers usually have to cut down regularly on the farm, so probably they will not remove all of the trees, obviously, because the plant would then be dead. Nevertheless, they can use the leaves during normal cutting on the plantation. This is really the expertise of the farmer. In addition, pruning leads to a lot of material. Therefore, even if farmers do the usual cutting, they can have a lot of coffee leaf tea. Then again, it should be considered that the time of pruning is independent of the moment when harvesting. It is not the same time of year and there are different moments when farmers can gain leaves. Moreover, it even increases the yield of the plant.

### 3.9. Can Pruned Leaves and Twigs Be Used for Compost Preparation and How Far Is Their Usage?

They can certainly be used for the preparation of compost preparation [40]. However, farmers must be aware of the increased concentration of caffeine they are bringing into the farm with this compost depending on the stage of degradation, risking autotoxicity effects diminishing production (see [7]).

### 3.10. Sensorially, How Would You Rate Cascara Beverages Compared to Coffee Bean-Based Ones?

This question is probably a bit difficult to answer because they are not comparable, because the materials are so different. Cascara has a fruity taste and could be very acidic. Startups pursuing pasteurization often guarantee 12-month stability at ambient temperature.

### 3.11. EFSA Has Proposed Tough Microbiological Criteria for Cascara. Do You Think They Can Be Met?

Yes, even without using additional procedures, it seems the specifications can be met. However, some farmers are spraying the cascara with alcohol and other agents during the drying process. These questionable processes must be checked for compatibility with the respective food laws.

### 3.12. Do You Think Non-Organic Cascara Is Possible Given Pesticide Problems? Or That Pesticide Issues Require Specific Supply Chains?

Yes, suppliers have to be aware that they are buying for food uses. Typically, suppliers are currently buying only organic cascara. However, even organic ones should be tested for pesticide residues.

### 3.13. Do You Recommend Making a Cascara Blend for Cascara like in Normal Coffee?

Currently, the typical practice is to make blends from different varieties, because farms are currently not conducting such a selection process. Exceptions may be found, for example, in Panama, where pure variety cascara is found, such as *C. arabica* var. Gesha, Typica, or Bourbon.

### 3.14. Are Parchment Flakes Commercially Available?

At this moment, the product is still on the research stage. An industrial solution for this by-product needs to be established.

### 3.15. What Would Be the Cost of Production of 1 Kg of Coffee Silverskin?

Currently, this cannot be estimated. In fact, the silverskin accumulation involves a cost for the roasters because they must discard them. However, if the silverskin becomes an interesting for the food industry, it could become a resource.

### 3.16. Is There Any Harm When Silverskin Is Used for Animal Feed?

The use of by-products of coffee by animals is limited due to their caffeine content, which diminishes the palatability and acceptance [41]. Some of the oldest references on coffee by-products also showed that they might have a negative effect on weight gain in monogastric animals. However, levels of 10% in standard feed were judged as being safe [2,3].

### 3.17. Are There Any Trials on Adding Coffee Silverskin as an Ingredient in Food Preparations, e.g., Packaged Food Items or Biscuits?

There are several food uses (see Section 2.7 above), such as in bakery products, biscuits, meat burgers, but also as an ingredient with smoke flavor (also see [7]). There are also uses in paper industry [42]. The critical issue is the content of heat-induced contaminants, such as acrylamide, which need to be controlled.

### 3.18. Do You Have Information on Toxicological Tests of Silverskin in Rats?

Indeed, there are several in vivo studies of coffee silverskin available in the literature. Although long-term or chronic studies are not currently available, the available studies did not raise safety concerns about the use of silverskin as food [43,44,45,46].

### 3.19. Does Coffee Silverskin Pose a Risk from Heat-Induced Contaminants Such as Acrylamide?

Probably not because the acrylamide content is comparable to that of bread. It is also below the limit for coffee and probably the consumption of silverskin would be lower or comparable. However, the acrylamide content is influenced by the roasting process and should be controlled. Depending on the applications in the future, other heat-induced contaminants should also be considered, but as is currently known, they are not formed in excessive concentrations compared to other foods.

### 3.20. What Are the Main Food Applications of Silverskin, and Where Could Its Flavor Be Used?

Taking into account the flavor, the use of coffee silverskin could be various foods and beverages (see Section 2.7). For example, tea with coffee silverskin can be made. Silverskin is ready to use because it is dry and stabile.

Non-food applications include the use of silverskin as a raw material for paper production [42]. However, for human consumption, it could be used, for example, in any type of bakery product, extruded food products or even meat products [28,47]. For example, it could be used in breads that would usually be covered with herbs or on the surface of potato breads [7].

Silverskin can be used as a flavoring compound. For instance, in spices or salts, silverskin exhibits a nice smoky character, and flavor. Unlike guaiacol-type smoke flavors, which remind of smoked ham or smoked fish, the silverskin flavor reminds of a firewood flavor, which may even be suitable for vegetarian or vegan consumers, who often dislike guaiacol-type smoke flavors. There are not many natural sources in the food industry for such alternative types of smoke flavor [7].

### 3.21. From An Economic Perspective, Will You Recommend a Farmer to Focus on a Single Food Application of the Plant Exclusively, e.g., as Cascara or Typical Coffee Beverage?

One cannot give a blanket answer to that. It depends very much on the farm, i.e., on the location of the farm and on the overall conditions of the farm. If it is very far away, it is probably going to take a lot of effort to do one or the other, and it will depend a little bit on the climate. One has more water, and the other does not have as much water. Now, if a lot of water is needed for certain processing styles, for example, that is going to be a crucial issue. Or how dry is it in the harvest season? Can farmers just air dry things? Or do they need large drum dryers, for example, so that the products do not mold.

In principle, it is of course tactically better if the risk is split, i.e., build up several different pillars because then farmers can always compensate with something else if one price is high or low or if one yield is good and the other is bad.

It will be a question of making a recommendation about whether farmers should only produce cascara, for example, or whether they should also produce coffee flowers, or whether they should distill, for example. Who should distill? First, of course, the person who is allowed to do it. In some countries, it would be forbidden to distill.

Therefore, it is a complex issue that always depends very much on the individual farm, i.e., on the production conditions, and what can be added without the greatest effort. If farmers wanted to make, as an absurd example, candied coffee blossoms, which are very expensive. However, it is incredibly time-consuming to make candied blossoms, and that will only be worthwhile for someone who has so much coffee and so many blossoms that it is also worthwhile to invest in such a system with which they can do it automatically. That is, for a small farm, such a purchase will simply not pay off and amortize. Alternatively, they can make air-dried coffee blossom tea out of it, but nothing else. Most of it will come down to business management considerations.

### 3.22. How Will Industry/Authorities Protect against Adulteration of Cascara, Especially in the Era of E-Commerce?

Currently, there is no substitute product that is even cheaper than cascara, so that counterfeiting would be worthwhile. Food adulteration always presupposes that the substitute is cheaper, but what is cheaper than something that is currently being thrown away? Criminals will always try to find a loophole and authorities will then again have to close it. Therefore, the completely normal cat and mouse game always exists.

It first must be learned how cascara is characterized and what suitable analytical methods are. Cascara will of course have a certain fingerprint in various laboratory analyses, depending on the different species and varieties. Probably some standardization is also needed, e.g., via ISO standards as established for other herbal products such as tea and spices.

The current problem with cascara is that suppliers may be dealing with impure products, which have a bad taste, or that may be contaminated with pesticides or copper because they have been sprayed.

## 4. Conclusions

Historic literature shows that the practice of using by-products as food was widely known in the 18th and 19th centuries (see review in [5]), but has been lost over the last 100 years when the focus on the bean as a commodity rather than as a food was shaped by international trade. Specifically, the fleshy fruit, which is suitable for consumption, as evidenced by some coffee-eating animal species [48], appears to be applicable for human nutrition as well, but also some of the other by-products such as parchment, silverskin, leaves or flowers.

As this conference session has confirmed, all of the coffee by-products do deliver a high level of economical extract, and added value to the farms. There are huge potentials for all of these products, especially on markets not currently accessible due to regulations.

The authors hope that this conference has stimulated interest and future research on coffee by-products. Hopefully, another conference dedicated to coffee by-products will be possible in the near future as an in-person meeting, when the COVID-19 pandemic has been overcome.

## Figures and Tables

**Table 1:** Coffee by-products and assessment of their novel food status in the European Union (EU) considering regulation (EU) No 2015/2283 (updated with permission from Klingel et al. [5] and Lachenmeier et al. [7]).

Coffee By-Product	EU Novel Food Status ^a^
Flowers (blossoms)	Novel, currently unapproved. Some anecdotal evidence for traditional food uses in a third country. Needs approval procedure.
Leaves	Authorization granted for infusion of coffee leaves based on notification as traditional food from a third country [8,9,10].
Coffee cherry materials (husks, cascara, dried or fresh coffee cherries, coffee pulp or mucilage)	Novel, currently unapproved. Notifications have been submitted for cascara and cherry pulp as traditional food from a third country [11,12] as well as a complete application for further uses [13]. Evaluations of the European Food Safety Authority are available [14,15].
Green unroasted beans	Not novel [16]. The classification also applies to non-selective water extraction made of them. Selective extracts could be novel.
Silver skin	Unclear but indirect consumption before 1997. Consultation currently in progress.
Coffee grounds	Not novel (spent coffee grounds, defatted spent coffee grounds and defatted unused coffee grounds) [17].
Stems, twigs, wood	Non-food material, contamination up to certain levels typically tolerated in the trade of green coffee.
Parchment	Novel, currently unapproved. No application pending. Needs approval procedure (possibly in connection with cascara from dry process, which contains parchment)

^a^ Authors’ judgment considering the EU Novel Food catalog [16], article 4 consultations and pending applications/notifications.

**Table 2:** Chemical composition of coffee silverskin from *Coffea arabica* and *Coffea canephora* as well as coffee silverskin pellets based on dry material (reprinted with permission from Gottstein et al. [6]).

Constituent	*C. Arabica* Silverskin	*C. Canephora* Silverskin	Silverskin Pellets
Dietary Fiber (g/100 g)	67.0 ± 1.0	62.0 ± 0.4	59.1 ± 0.02
Insoluble (g/100 g)	56.0 ± 0.4	53.2 ± 0.3	46.0 ± 0.2
Soluble (g/100 g)	11.0 ± 1.7	8.8 ± 0.5	13.1 ± 0.2
Fat (g/100 g)	1.57 ± 0.03	1.50 ± 0.02	1.82 ± 0.04
Ash (g/100 g)	8.15 ± 0.08	9.50 ± 0.14	11.24 ± 0.01
Protein (g/100 g)	18.1 ± 0.2	22.2 ± 0.5	17.8 ± 0.1
Caffeine (g/100 g)	0.80 ± 0.002	0.86 ± 0.03	0.76 ± 0.01
Acrylamide (µg/kg)	152	161	24
5-Hydroxymethylfurfural	pos. (<LOQ)	pos. (<LOQ)	pos. (<LOQ)
Furfuryl alcohol	n.d.	n.d.	n.d.
Moisture content (%)	6.15 ± 0.12	6.57 ± 0.06	7.64 ± 0.02

LOQ, limit of quantification; n.d., not detectable; *n* = 3 for caffeine; *n* = 2 for dietary fiber, fat, ash, protein, 5-hydroxymethylfurfural, and furfuryl alcohol; *n* = 1 for acrylamide; relative standard deviation 15% for acrylamide.

## Data Availability

No new data were created or analyzed in this study. Data sharing is not applicable to this article.
